# EFFECTS OF COLD ISCHEMIA TIME ON HEPATIC ALLOGRAFT FUNCTION

**DOI:** 10.1590/0102-6720201700040003

**Published:** 2017

**Authors:** Alexandre Coutinho Teixeira de FREITAS, Desirée de Marillac Nascimento de MATOS, Jorge Amilton Tosato MILSTED, Julio Cezar Uili COELHO

**Affiliations:** 1 Clinical Hospital Complex, Hepatic Transplantation Service; 2 Faculty of Medicine, Federal University of Paraná, Curitiba, PR, Brazil

**Keywords:** Liver transplantation, Cold ischemia, Allografts, Transplante de fígado, Isquemia fria, Aloenxertos

## Abstract

*****Background*** ****:**

Cold ischemia time is related to success of liver transplantation.

*****Aim*** ****:**

To compare the impact of cold ischemia time on allografts locally collected to those collected distantly.

*****Methods*** ****:**

Were evaluated 83 transplantations. The patients were divided in two groups: those who received liver grafts collected from cities out of Curitiba (n=42) and locally (n=41). From the donors were compared: cause of death, days at ICU, cardiac arrest, vasoactive drugs, lab exams, gender, age, and BMI. Were compared the subsequent information of receptors: cold ischemia time, warm ischemia time, length of surgery, lab exams, etiology of cirrhosis, MELD score, age, gender, histology of graft, use of vasoactive drugs, and blood components transfusion. Were evaluated the correlation between cold ischemia time and lab results.

*****Results*** ****:**

The liver grafts collected from other cities were submitted to a longer cold ischemia time (500±145 min) compared to those locally collected (317,85±105 min). Donors from other cities showed a higher serum sodium level at donation (154±16 mEq/dl) compared to those from Curitiba (144±10 mEq/dl). The length of cold ischemia time was related to serum levels of ALT and total bilirubin.

*****Conclusion*** ****:**

Liver grafts distantly collected underwent longer cold ischemia times, although it caused neither histologic injuries nor higher transfusion demands. There is a correlation between cold ischemia time and hepatic injury, translated by elevation of serum ALT and total bilirubin levels.

## RESUMO

*****Racional*** ****:**

O tempo de isquemia fria está relacionado ao sucesso do transplante hepático.

*****Objetivo*** ****:**

Comparar o impacto do tempo dela sobre enxertos captados localmente com os distantes.

*****Métodos*** ****:**

Avaliaram-se 83 transplantes. Os pacientes foram divididos em dois grupos: enxertos captados fora de Curitiba (n=42) e captados localmente (n=41). Dos doadores compararam-se causa do óbito, dias de UTI, parada cardíaca, drogas vasoativas, exames laboratoriais, gênero, idade e IMC. Dos receptores seguintes dados: tempos de isquemia fria e morna, tempo operatório, exames laboratoriais, causa da cirrose, MELD, idade na operação, gênero, biópsia do enxerto, uso de drogas vasoativas e necessidade de transfusões. Foi realizada avaliação de correlação entre o tempo de isquemia fria e os exames laboratoriais.

*****Resultados*** ****:**

Os enxertos captados à distância foram submetidos a maior tempo de isquemia fria (500,3±145 min) quando comparados aos captados localmente (317,85±105 min). Os doadores de fora apresentaram níveis mais elevados de sódio no momento da doação (154±16 mEq/dl) comparados aos doadores de Curitiba (144±10 mEq/dl). Houve correlação entre o tempo de isquemia fria e os níveis de ALT e de bilirrubina total. Não houve diferenças ao comparar-se os demais dados.

*****Conclusão*** ****:**

Enxertos captados à distância sofreram maior tempo de isquemia fria. Isso não refletiu nos prejuízos histológicos nem na demanda transfusional durante o pós-operatório. Houve correlação entre o tempo de isquemia fria e o grau de lesão hepática avaliada pela ALT e pela bilirrubina total.

## INTRODUCTION

The first liver transplantation in humans was done in the 60s by Thomas Starzl. Since that time to the current days several landmarks have been conquered improving surgical technique, ameliorating grafts preservation during transportation, controlling cellular rejection, and preventing infections on post-transplantation period^19^ ^,^ ^20^ ^,^ ^22^. 

In Brazil, liver transplantation has conquered wide acceptance by medic community and is today the surgical modality of choice to treat hepatic diseases in advanced stages, allowing great improvement in quality of life and lifespan of patients submitted to this technique.

In medical literature, many variables unrelated to surgical technique have been stablished as transplant`s failure risk factors, including features of donors and inherent factors of receptors. Of those elements, reducing cold ischemia time can be potentially managed by transplant’s team aiming to minimize its deleterious effects over the graft. Cold ischemia time comprises the period since the clamping of donors’ vessels and infusion of cold preservation solution, and, therefore, loss of blood supply to the liver, until the moment in which the graft is inserted into the abdominal cavity of the receptor. During this period, the liver is found perfusioned by preservation solution and maintained in hypothermic conditions to minimize the ischemic suffering.

Cold ischemia time is directly influenced by the need to transport the organ from the donor to the recipient. In many cases long distances have to be traveled, especially when transportation is done between cities located far away. Additionally, the logistics demand efficient communication between harvesting and transplantation teams. Defining a safe cold ischemia time interval has being a matter of discussion in several studies. In the state of Paraná, it is responsibility of the transplantation team to analyze individually the possibility of using a liver graft offered by the State Agency that regulates this matter (Transplantation State Agency).

Amongst the spectrum of transplant’s prognostic risk factors, graft failure has been the main worry once it is associated to high morbidity and mortality^13^. Prolonged cold ischemia has been pointed as an independent risk factor of graft’s acute rejection^23^. It also correlates to an increase in post-transplantation mortality^6^. This susceptibility to ischemia is increased even more when added of other risk factors from the cadaveric donor, as: age, cardiac arrest previously to harvesting, and usage of vasoactive drugs. Risk factors from the receptor such as MELD score and demand for blood transfusion are also important^4^ ^,^ ^13^. A previous study performed in Pittsburgh, USA, evaluated the influence of distance between harvest and transplant location site and the cold ischemic time in the prognosis of liver transplantation. It concluded that far distances between those cities increases significantly the cold ischemia time which the graft is submitted, and also evidenced direct relation between cold ischemia time and frequency of primary dysfunction and loss of graft^21^. 

The purpose of this study was to, comparatively, analyze the effects of cold ischemia time on locally harvested livers, in Curitiba’s metropolitan area, to those distantly harvested in another city or state. 

## METHODS

The study analyzed medical records of patients submitted to liver transplantation at Hospital de Clínicas of Federal University of Paraná, Curitiba, PR, Brazil between 2006 and 2015. This period corresponds to the era after implantation of MELD score as a rule to allocate grafts on waiting list for transplantation in Brazil^5^. Data from organs locally and distantly harvested were analyzed. Transportation and city of origin were assessed in the records of Transplantation State Agency.

Were included only receptors that had an age equal or above 18 years old at surgery and underwent conventional liver transplantation with reconstruction of inferior vena cava. Were excluded pediatric patients, cases of inferior vena cava’s conservation (piggy-back technique), insufficient data records, and decease at surgical room or at immediate postoperative hour.

The patients were divided into two groups and compared accordingly to the place of graft harvesting: those from Curitiba’s metropolitan area and those from cities located far away. Cold ischemia time was stated as the interval since aortic clamping and infusion of cold preservation solution in the donor’s graft until insertion into receptor’s abdominal cavity. Warm ischemia time was defined as the interval from insertion of the graft to detachment of vascular clamps after finishing all venous anastomosis (suprahepatic and infrahepatic inferior vena cava and portal vein). Both cold and warm ischemia times were compared between groups. Furthermore, was evaluated whether there was correlation of cold ischemia time to bilirubin values, alkaline phosphatase, gamma-GT, ALT, AST, sodium, creatinine, albumin and RNI without splitting groups.

The following parameters were also analyzed in the donors and compared between both groups: gender, age, body mass index, days at critical care unit, usage of vasoactive drugs, cardiac arrest previous to harvesting, AST, ALT, sodium, creatinine and cause of decease. Was computed only the last blood test done previously to organ’s harvest.

In the receptors were compared the following data: gender, age at surgery, MELD, cirrhosis’ etiology, length of procedure, cold ischemia time, warm ischemia time, usage of vasoactive drugs on 1^st^ postoperative day, transfusion of packed red blood cells, platelets, and fresh frozen plasma, AST, ALT, RNI, alkaline phosphatase, gama-GT, sodium, creatinine, total bilirubin and fractions, albumin, and levels of ischemia and steatosis signs on graft biopsy at the end of transplantation. Were evaluated only the samples collected within 8 and 24 h after surgery. Only transfusions within first 24 h since intensive care unit admission were deemed.

Protocol grafts’ biopsies collected surgically at the end of implantation, before abdominal cavity synthesis, underwent anatomopathological examinations. Ischemic findings and fatty infiltration were compared between groups. Ischemic findings were classified according to microscopic description as substantial, mild and subtle. Zone 3 necrosis was considered important and in different degrees: as moderate when hepatocytic balloonization with initial autolysis was present, and as discrete when hepatocyte swelling without autolysis existed. Steatosis was classified in grades 1 and 2 according to the NAS score^14^.

### Statistical analysis

Mann-whitney test was used on evaluation of results expressed in numeric values, Fisher’s exact and chi-square tests to compare proportions, and Spearman’s to correlate cold ischemia time with findings.

## RESULTS

Were initially assessed 125 cadaveric liver transplants. Forty two were excluded: 16 underwent inferior vena cava preservation (piggy-back technique); in eight data records were not found on Transplantation State Agency; in seven data records were not found on hospital’s archives; seven had no biopsies; three per-operative deceased within first hour; and one transplant de novo. Eighty three patients were included, 61 males and 22 females, average of 52 years old (Table 2). Forty-one had grafts harvested in Curitiba’s metropolitan area and 42 collected distantly. The average cold ischemia time were 317 min in those harvested in Curitiba. This value was shorter than the average 500 min observed on those distantly collected (p<0,0001).

**TABLE 1:** Epidemiologic and clinic aspects of donors: harvests in Curitiba vs. other cities

Donors	Curitiba	Other cities	p
	n=41	n=42	
Gender (M/F)	20/21	23/19	NS
Age	36.5 ± 15 (11-65)	33.8 ± 15.1 (9-63)	NS
BMI kg/m2	24 ± 2.8	25.2 ± 3.2	NS
Days in ICU	4.51 ± 3.53 (1-16)	4.9 ± 3.15 (1-13)	NS
VAD (Y/N)	35/6	36/6	NS
CA (Y/N)	9/31	3/39	NS
AST (U/dl)	88.4 ± 125.4	71.25 ± 81	NS
ALT (U/dl)	60.71 ± 51	49.3 ± 46.3	NS
Na (mEq/dl)	144.7 ± 10.6	154 ± 16.1	0.0034*
Cr (mg/dl)	1.28 ± 1.31	1.19 ± 0.65	NS
Causa mortis	CET = 18	CET = 24	NS
	hCVA = 14	hCVA = 16	
	iCVA = 4	iCVAi = 1	
	CA = 3	CA = 1	
	CVST = 2		

Quantitative data expressed in mean±standard deviation (variation); Qualitative data correspond to info in brackets. NS= nonsignificant; BMI= body mass index; VAD= vasoactive drugs; CA=cardiac arrest; AST= aspartate aminotransferase; ALT= alanine aminotransferase; Na= serum sodium; Cr= serum creatinine; CET= cranioencephalic trauma; hCVA= hemorrhagic stroke; iCVA= ischemic stroke; CVST= venous stroke

**TABLE 2:** Preoperative variables of receptors: harvests in Curitiba vs. other cities

Receptors	Curitiba n=41	Other cities n=42	p
Gender (M/F)	30/11	31/11	NS
Age	53 ± 8 (24-70)	51 ± 10 (22-70)	NS
MELD	17.3 ± 5.66 (7-40)	16.7 ± 5.99 (8-37)	NS
Etiology of cirrhosis			
Hepatitis C	12	11	NS
Alcoholic cirrhosis	10	11	NS
Hepatocellular carcinoma	8	13	NS
Hepatitis B	5	9	NS
Cryptogenic Cirrhosis	5	4	NS
Auto-immune Hepatitis	4	3	NS
Others	5	7	NS

Quantitative data expressed in mean±standard deviation (variation); diseases classified in “others: are: drug- induced hepatitis, drug-induced fulminant hepatic failure, non-alcoholic steatohepatitis, primary sclerosing cholangitis, primary and secondary biliary cirrhosis, Budd-Chiari syndrome, alfa-1-antitripsine deficiency, and cirrhosis due to hemochromatosis

The average level of serum sodium was 144 mEq/dl on those grafts locally harvested, lower than the 154 mEq/dl that found when the liver was collected far away (p=0.0034, Table 1). No differences were observed on all remaining parameters (Table 1). Donors in Curitiba were 36.5±15 years old, 20 males and 21 females. The mean BMI was 24±2.8 kg/m^2^. They stayed at intensive care unit 4.51±3.53 days; 85% of them required vasoactive drugs. Nine suffered cardiac arrest previously to harvesting (Table 1). All lab exams are expressed on Table 1. Donors from other cities were in average in 33.8±15 years old, 23 males and 19 females, with a BMI of 25.2±3.2 kg/m^2^. They stayed at intensive care unit 4.9±3.15 days, 84,5% required hemodynamic support. Three suffered cardiac arrest (Table 1).

The sample was homogeneous in relation to causa mortis (Table 1). Cranioencephalic trauma was the main cause of death in both groups, followed by hemorrhagic stroke, ischemic stroke, cardiac arrest and lastly venous stroke.

Receptors also constituted a homogeneous group in relation to gender, age, MELD and cirrhosis’ etiology (Table 2). The group that received grafts from Curitiba had a mean age of 53±8 years, 30 males and 11 females, and an average MELD score of 17.3±5.66. The other group, those who received grafts from cities far away, had 51±10.3 years, 31 males and 11 females, and a mean MELD score of 16.7±5.9.The sum of etiologies of cirrhosis on Table 2 does not match with the total of patients due to overlap of etiologies in 10 patients of Curitiba’s group and 17 of other cities’ group. Hepatitis C was present in 12 patients of first group, five of them also had hepatocellular carcinoma, one with both conditions plus hepatitis B. Similarly, hepatocellular carcinoma was diagnosed in three out of five patients with hepatitis B, including the patient with three conditions, and in two with alcoholic cirrhosis. On the second group, hepatocellular carcinoma was present in six of 11 patients with hepatitis C, in six of nine of hepatitis B, including one co-infected, and in two cases of alcoholic cirrhosis, one of them also with hepatitis C.

Table 3 shows data related to the transplantation process and laboratorial data of 1^st^ postoperative day. Both groups were similar on mean warm ischemia time, (56±18 min and 55±17 min), on mean surgery length (6.6±1.7 h and 6.3±1.7 h), on need of vasoactive drugs (18 in each), and blood transfusions need. There were none significant statistical differences in laboratory results between groups.

**TABLE 3:** Data about transplant and 1st postoperative: harvests in Curitiba vs. other cities

	Curitiba n=41	Other cities n=42	p
Length of surgery (h)	6.6 ± 1.7 (3.8-11)	6.3 ± 1.7 (3.75-10.1)	NS
Warm ischemia time (min)	56 ± 18.67 (30-120)	55.3 ± 17 (25-120)	NS
Vasoactive drugs (S/N)	18/23	18/24	NS
Transfusion (units)			
Red blood cells	2 ± 2.7 (0-12)	2.38 ± 5 (0-30)	NS
Fresh frozen plasma	1.65 ± 3 (0-10)	2 ± 3.3 (0-12)	NS
Platelets	1.3 (0-20). ± 3.8	1.6 (0-10). ± 3.1	NS
Laboratory:			
AST (U/l)	1529 ± 1653 (123-9872)	2506 ± 3282 (312-14700)	NS
ALT (U/l)	915 ± 869 (125-4342)	1071 ± 930 (195-4390)	NS
INR	1.83 ± 0.83 (0.97-5.43)	2.2 ± 1.48 (1.16-10)	NS
Alkaline phosphatase (U/l)	142 ± 152 (36-919)	96 ± 51 (30-241)	NS
Gama-GT (U/l)	133 ± 130 (30-578)	105 ± 80 (28-505)	NS
Sodium (mEq/dl)	140.4 ± 4.4 (131-151)	139.7 ± 6.2 (123-156)	NS
Creatinine (mg/dl)	1.17 ± 0.56 (0.5-3.9)	1.4 ± 0.81 (0.6-4.3)	NS
Total bilirubin (mg/dl)	3.81 ± 2.2 (0.42-8.23)	3.89 ± 2.5 (0.59-13.65)	NS
Direct fraction (mg/dl)	2.5 ± 1.6 (0.37-5.72)	2.6 ± 1.58 (0.34-7)	NS
Indirect fraction (mg/dl)	1.2 ± 1 (0.15-5.13)	1.1 ± 0.73 (0.1-3.72)	NS
Albumin (g/dl)	2.4 ± 0.5 (1.3-4.1)	2.3 ± 0.5 (1.0-3.8)	NS
Histopathology of graft:			
Ischemia (Y/N)	22/19	25/17	NS
Substantial (Y/N)	0/41	2/40	NS
Mild (Y/N)	7/34	7/35	NS
Discreet (Y/N)	15/26	16/26	NS
Steatosis (Y/N)	12/29	11/31	NS
Grade I (Y/N)	8/33	9/33	NS
Grade II (Y/N)	3/38	2/40	NS

Quantitative data expressed in mean±standard deviation (variation); NS=non significant; AST=aspartate aminotransferase; ALT=alanine aminotransferase; INR= International Normalized Ratio of prothrombin; Gama-GT= gamma-glutamyl-transferase

**FIGURE 1:**
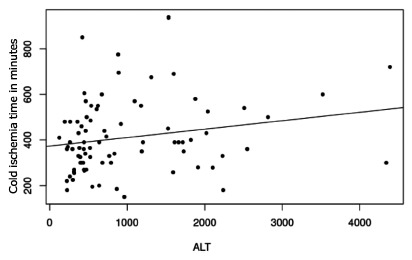
Plot of correlation of cold ischemia time and ALT (p=0.02)

**FIGURE 2:**
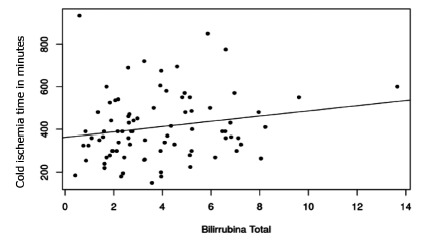
Plot of correlation of cold ischemia time and total bilirubin (p=0.05)

Spearman’s test between cold ischemia time and the various laboratorial results in all patients demonstrated significant direct correlation between length of cold ischemia time and rising of ALT serum levels (p=0.02, Figure 1). It also showed a direct correlation to total bilirubin (p=0.05, Figure 2).

## DISCUSSION

Cold ischemia time has been related to liver transplantation success because its an important factor of allograft damage. Decrease in ATP level, increase in free radicals production, release of cytokines, cellular dysfunction, and apoptosis are sequential events promoted by the ischemic suffering process to which the graft is subjected^16^. The intensity of tissue injury arising as a result of cold ischemia time is reflected laboratorially by the elevation of liver enzymes, abnormal results on graft biopsy, and demand for vasoactive drugs and transfusions to sustain hemodynamic stability during the postoperative period^2^.

An optimal cutoff for cold ischemia time duration remain undefined and it is subject of extensive discussion. Some transplantation groups have recommended 12 h as the maximum duration of cold ischemia time to preserve allograft function. Other recent studies suggest that this matter may be subjected to individualization of each patient based on other risk factors present at the moment of the surgery^1^ ^,^ ^10^. Consequently, a safe cold ischemia time is variable between different transplantation groups and according to the studied population. Regional studies on the impact of cold ischemia time are required.

Since factors related to liver donors may impact on graft postoperative function, was evaluated the presence of homogeneity concerning donors data from several variables. The two groups analyzed showed homogeneity regarding age, gender, anthropometric data (height, weight, BMI), cause of death, serum levels of AST, ALT and creatinine, as well as preceding demand for vasoactive drugs and occurrence of cardiorespiratory arrest before donation. Only a single laboratory data from donors was discrepant between the two groups: the serum sodium level was higher in the group with organs harvested distantly. The hypernatremia reflects a common physiological alteration present in patients after encephalic death. According to previous studies, hypotension, hypothermia, hypernatremia and insipid diabetes are common findings in potential organ donors^9^. Such physiological conditions imply the need for constant correction of these variables by ICU professionals. In the present study, hypernatremia detected in such donors may be one evidence reflecting the poor quality of donor physiological maintenance in intensive care units from cities outside the capital. However, there is evidence supporting that hypernatremia alone does not have any negative impact on graft outcome^6^ ^,^ ^7^ *.*

Concerning the homogeneity between all 83 recipients evaluated, the main causes that determined indication for liver transplantation were hepatitis C followed by alcoholic cirrhosis. Such proportions were similar between the two groups analyzed and are in agreement with those described in literature^18^. Other recipient factors influencing on the prognosis of the transplant, such as the age of the recipient and the MELD score, did not show discrepancies^12^. The gender of the patients had the same proportions. Additionally, the mean duration of transplantation and the warm ischemia time were similar between the two groups, as expected, since operations were performed by the same transplantation team. This circumstance was fundamental to evaluate the cold ischemia time without the functional interferences offered by the warm ischemia time, another important inducer of tissue damage.

Liver grafts distantly harvested underwent longer cold ischemia. This finding is caused by the distance between the services on the time of transportation. The sample comprised donations from several cities in the state of Paraná, as well as two organs from the state of Santa Catarina and one from Rio Grande do Sul. Accredited liver transplantation centers in Paraná totalize six hospitals, all of them are located within the metropolitan region of Curitiba. Thus, a good functioning of the organ distribution system is an essential premise for minimizing the cold ischemia time to which liver grafts are subjected during the displacement from other cities. In our sample, the maximum cold ischemia time was 16 h.

Prolonged cold ischemia time evidenced in the present study caused neither significant differences in histology nor increased the transfusional demand during the first postoperative day. However, there was a clear relationship between cold ischemia time and elevations in serum levels of two liver function markers: ALT and total bilirubin. These elevations suggests the presence of hepatic ischemic injury^11^. In fact, alanine aminotransferase has been described as an enzyme that is closely related to hepatocellular damage and is also associated with prognosis and mortality after orthotopic liver transplantation^15^. ALT is present in very low concentrations in extrahepatic sites, a fact that translates its serum elevation as the most specific laboratory marker for liver injury^8^ ^,^ ^11^. In contrast, the presence of hyperbilirubinemia is associated with cholestatic injury. Literature describes a correlation between serum bilirubin elevation and higher rejection rates and propensity for infections during post-transplant period^17^. The pathogenesis of cholestasis after orthotopic transplantation has been explained by several factors, including the duration of cold ischemia time3.

No correlation was found between cold ischemia time and serum levels of other cholestatic enzymes (gamma-GT and alkaline phosphatase) nor AST. This might be explained based on the fact that it is a retrospective study and by the size of data collected. Retrospective studies should have a cautiously interpretation of results and then a posterior validation by prospective series. However, the strong association between cold ischemia time and laboratory levels of ALT and total bilirubin found in the present study was relevant to patients included in our data.

## CONCLUSION

Cold ischemia time from distantly harvested grafts was longer compared to grafts captured within the metropolitan region of Curitiba. The mean serum sodium level was higher in donors of organs harvested distantly. A correlation between cold ischemia time and hepatic injury was observed, translated by elevation of serum ALT and total bilirubin levels. In patients undergoing liver transplantation, the prolongation of cold ischemia time does not produce histological changes, neither show higher demands for transfusion or vasoactive drugs, nor change laboratory parameters evaluated during the first 24 h postoperative time.
